# Developing and validating a scale for measuring pre-service Chinese as an additional language teacher beliefs

**DOI:** 10.3389/fpsyg.2022.989581

**Published:** 2022-09-14

**Authors:** Chili Li, Ting Yi, Shuang Zhang, Chunyan Ma, Honggang Liu

**Affiliations:** ^1^School of Foreign Languages, Hubei University of Technology, Wuhan, China; ^2^Research Institute of International Chinese Language Education, Beijing Language and Culture University, Beijing, China; ^3^School of Humanities, Wuhan University of Engineering Science, Wuhan, China; ^4^Department of Education, Faculty of Arts and Social Sciences, New Era University College, Selangor, Malaysia; ^5^School of Foreign Languages, Soochow University, Suzhou, China

**Keywords:** pre-service teachers of Chinese as an additional language, teacher beliefs, scale development and validation, exploratory factor analysis, confirmatory factor analysis

## Abstract

Teacher beliefs are a pivotal psychological quality for sustainable teacher development. Previous studies have mainly focused on the beliefs of English-as-a-second/foreign-language (ESL/EFL) teachers, while little attention has been paid to those of Chinese-as-an-additional-language (CAL) teachers. Particularly, there is a paucity of effort made to develop and validate instrument for measuring pre-service CAL teacher beliefs. Therefore, to further quantify the beliefs of CAL teachers is increasingly called for as an essential means to help teachers sensitize their beliefs system and promote teacher development as a sustainable goal. To be specific, the present study aims to construct a scale for gauging beliefs of pre-service CAL teachers. It firstly conceptualizes the dimensions of pre-service CAL teacher beliefs by means of semantic analysis with ROST CM6, and then cross-validates the reliability and validity of the scale with psychometric methods. Two independent samples composed of 221 and 222 pre-service CAL teachers participated in a questionnaire survey. The two samples were utilized for later Exploratory Factor Analysis (EFA) and Confirmatory Factor Analysis (CFA), respectively. The two data sets have satisfactory psychometric results, all confirming that the scale subsumes three factors: *Beliefs about Chinese Language Teaching (BCLT)*, *Beliefs about Chinese Language (BCL)*, and *Beliefs about Chinese Language Learners (BCLL)*. The scale validated in the present study contributes to research on pre-service CAL teacher beliefs, and provides implications for sustainable pre-service CAL teacher training.

## Introduction

Teacher beliefs are of critical importance to sustaining the development of pre-service second language (L2) teachers (Chan and Gao, 2014; Clark-Goff and Eslami, 2016), and have been a prevailing hotspot in the past decades (Xu, 2012; Portolés and Martí, 2020). Specifically, beliefs may promote pre-service teachers’ reflection on their teaching practice (Çapan, 2014; Vosniadou et al., 2020) and shape their decisions in accepting new teaching approaches, techniques, and activities (Cross, 2009; Xu, 2012). Exploring pre-service L2 teacher beliefs contributes to crystallizing the criteria to measure the sustainable professional growth of teachers (Altan, 2012; Tsunemoto et al., 2020). It is thus of paramount significance to examine pre-service teacher beliefs, particularly pre-service Chinese-as-an-additional-language (CAL) teacher beliefs considering the increasing demand for CAL teachers due to the rising number of CAL learners throughout the world (Wang and Zhang, 2021).

The number of CAL learners has exceeded 20 million by the end of 2020 (Li et al., 2021; Sun, 2021). Accordingly, the incremental population of Chinese language learners has triggered a worldwide demand for CAL teachers (Wang and Du, 2014). However, the outbreak of COVID-19 hazarded the international mobility of CAL learners and thus reduced the volume of the CAL population (Gao and Zhang, 2020). The volume of CAL teachers appointed abroad by the Center for Language Education and Cooperation of China also considerably withered (Ding, 2021). These undesirable changes might exert negative influences upon the beliefs of pre-service CAL teachers. Therefore, how to sustain and strengthen positive beliefs about language teaching among teachers, pre-service teachers in particular, against the adversary times has become a critical issue (Karataş and Tuncer, 2020; Yang et al., 2021). It is unknown how the pre-service CAL teachers view the teaching of Chinese to those learners from diversified linguistic, cultural, educational, and social backgrounds (Gong et al., 2021; Hua and Gao, 2021).

Existing research has mainly focused on pre-service English as a second/foreign language (ESL/EFL) teacher beliefs (Jaimes, 2013; Çapan, 2014). However, there is a dearth of research on pre-service CAL teacher beliefs. Present research on CAL teacher beliefs primarily adapts instruments which are developed for examining ESL/EFL teacher beliefs, such as the Grammar Teaching Belief Scale for Pre-service CAL Teachers (Ji and Liu, 2012) and the Volunteers of CAL Teachers’ Belief Scale (Song, 2018). There are to date few scales developed and validated for exploring pre-service CAL teacher beliefs. In response to the shortage of scales for measuring CAL teacher beliefs, Zhu (2019) develops a scale with multiple dimensions to measure CAL teachers’ beliefs. However, this study is only a preliminary design; it does not report the validity and reliability of the scale. Therefore, the present study attempts to construct a PCALTBS, and to empirically test its reliability and validity.

In summary, the existent scales targeted for CAL teacher beliefs are mostly adapted from the ones originally designed for exploring beliefs of ESL/EFL teachers. These scales may be illuminative for understanding the features of beliefs of the ESL/EFL teachers. However, the discrepancies between Chinese and English may undermine the applicability and appropriateness of these scales into measuring CAL teacher beliefs (Ma et al., 2017; Gong et al., 2020a). Another issue regards that the limited number of existent scales have not undergone rigid reliability and validity tests (Zhao, 2018; Bao et al., 2021). Therefore, in order to bridge these gaps, the present study attempts to develop and validate a PCALTBS, which is expected to enrich literature on CAL teacher beliefs and to shed light on CAL teacher training for sustainable teacher development.

## Literature review

### Pre-service second language teacher beliefs

Second language (L2) teacher beliefs have been extensively defined. The construct has been regarded as teachers’ practical knowledge (Portolés and Martí, 2020), self-efficacy and content-specific beliefs (Kagan, 1992), beliefs about language learning and teaching (Chu et al., 2021), and attitudes toward teacher-student relationship (Zembylas, 2005; Borg, 2011). Of these early efforts, Borg (2001) expounds teacher beliefs by structuring the construct into teachers’ beliefs about teaching, learning, learners and subjects. Borg’s (2001) classification has been embraced by most researchers (i.e., Karimi and Nazari, 2017; Song, 2018; Karaca and Uysal, 2021). The present study hence proposes to follow Borg’s (2001) definition and classification.

Present research mainly centers on teaching beliefs of ESL/EFL teachers (Karaca and Uysal, 2021). Specifically, these studies focus on ESL/EFL teachers’ beliefs about teaching English listening (Karimi and Nazari, 2017), speaking (Bai and Yuan, 2018; Farrell and Yang, 2019), reading (Kuzborska, 2011), writing (Yigitoglu and Belcher, 2014; Karaca and Uysal, 2021) and grammar (Andrews, 2003). These efforts pay considerable attention to the beliefs of language skills teaching, without examining teacher belief which is a complex and comprehensive system (Kagan, 1992). These early studies offer an in-depth understanding of ESL/EFL teacher beliefs, but little is known about beliefs of CAL teachers. It remains under-examined whether the features of ESL/EFL teacher beliefs could be applicable to unveiling those of pre-service CAL teachers.

### Pre-service Chinese as an additional language teacher beliefs

Efforts have been recently made to understand CAL teacher beliefs. Existing research primarily focuses on domain-related teaching beliefs of CAL teachers, such as beliefs about teaching grammar (Ji and Liu, 2012; Qian, 2017) and vocabulary (Shui, 2016) among the CAL teachers. For instance, Zhao (2018) divides CAL teacher beliefs into *BCL*, Chinese language teaching, Chinese language learning, Chinese cultural communication, and teacher-student relationship. However, she only proposes the analytical framework without offering empirical evidence. In addition, Song (2018) includes other dimensions such as Chinese language learner, testing and professional development into her taxonomy of CAL voluntary teacher beliefs. Similarly, Zhu (2019) proposes a five-dimension model of CAL teacher beliefs involving *BCL*, teaching, learning, learning environment, and professional development. These studies generate insight into the multi-sourced nature of CAL teacher beliefs. Nevertheless, they are not subjected to cross validation by means of exploratory and CFA.

The review of the relevant literature reveals a dominance of attention paid to the beliefs of in-service CAL teachers, but an inadequacy to pre-service CAL teacher beliefs. Inexperienced novice language teachers may have inappropriate or unrealistic beliefs about language teaching and learning (Altan, 2012). These concepts may be passed to students directly or indirectly through their teaching practice. Given the complexity of teacher beliefs, it is not only of titanic importance to examine the beliefs of in-service CAL teachers, but also of particular significance to explore those of pre-service CAL teachers.

### Existing measurements for assessing Chinese as an additional language teacher beliefs

Quantitative techniques such as questionnaire have been widely utilized in research on teacher beliefs (Altan, 2012; Cota Grijalva and Barajas, 2013). A number of representative measurements include Horwitz’s (1985) Beliefs about Language Learning Inventory (BALLI) and Andrews’ (2003) Teacher Belief Scale for English Learning. Horwitz’s (1985) BALLI scale is mainly directed to measure learners’ beliefs about language learning. She opens a window for understanding the divergences and convergences in beliefs about language learning and teaching between teachers and students. However, most of the BALLI items pertain to learners, instead of teachers (Karaca and Uysal, 2021). This thus makes it inappropriate to measure teacher beliefs. Another problem of the BALLI is associated with the reliability and validity of the scale. It has not been subjected to strictly test its validity and reliability (Nikitina and Furuoka, 2006; Ahn and Yang, 2009).

Andrews’ (2003) Teacher Belief Scale for English Learning draws much attention from the academia (Karaca and Uysal, 2021). The scale consists of six dimensions: Belief about English teaching methods based on form/accuracy, Belief about the use of deductive methods, Belief about inductive methods, Belief about communication/meaning-based teaching methods, Belief about the value of practice/rote memorization, and Belief about the importance of using metalanguage. This scale is subjected to verification of correlation between its components, which indicates good internal validity. However, its internal consistency is not tested by means of psychometric techniques such as exploratory factor analysis (EFA) and CFA.

In addition to the above scales, other instruments are also developed to measure CAL teacher beliefs. For instance, Zhu (2019) proposes a scale for measuring CAL teacher beliefs. The scale encompasses five dimensions in relation to *BCL*, Chinese language teaching, Chinese language learning, Chinese language environment, and Chinese language teacher development. This scale is elaborate in constructing the dimensions. However, it does not receive any test of validity and reliability. Sun and Zhang (2021) develop an instrument to measure pre-service CAL teachers’ beliefs about educational technology acceptance. It is based on Davis’ (1986) Technology Acceptance Model (TAM). This scale is subjected to exploratory and CFA, which can effectively prove its high reliability and validity. However, it only inquires CAL teacher beliefs about the integration of technology into teaching, which does not reveal the whole picture of CAL teacher beliefs.

In a nutshell, existing scales offer insightful implications for further exploring the dispositions of beliefs held by CAL teachers. However, these scales are mainly adapted from those designed to measure ESL/EFL teacher beliefs. They either lack inclusivity considering the complexity of the teacher beliefs system, or bear inadequate confirmation of validity and reliability. Therefore, more efforts are needed to develop and validate such an instrument for measuring pre-service CAL teacher beliefs.

## The current study

This study is segmented into two phases. At Phase One, a scale was constructed and administered to 221 student teachers majoring in *Teaching Chinese to Speakers of Other Languages* (TCSOL). Then, EFA was conducted on the collected data for the purpose of identifying the potential dimensions. At Phase Two, another sample composed of 222 TCSOL students responded to the same instrument of the questionnaire scale. This data set was subsequently processed by means of CFA so as to testify the validity and reliability of the scale.

### The questionnaire and its development process

**FIGURE 1:**
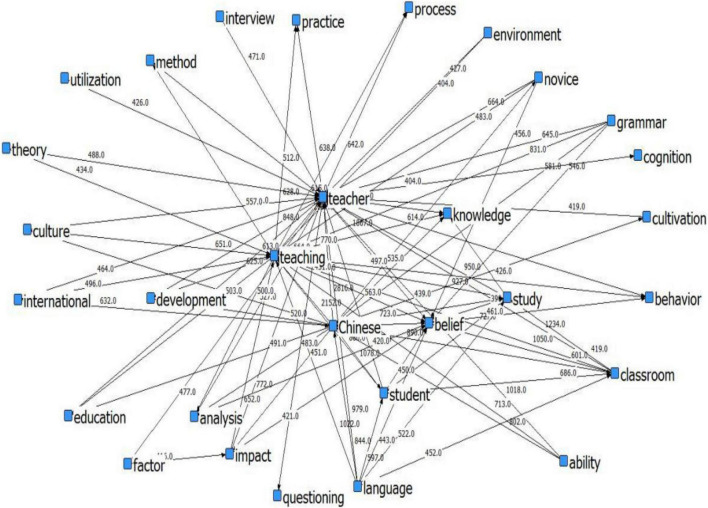
Semantic network of high frequency words.

Twenty two documents regarding beliefs of Chinese language teachers were retrieved from China National Knowledge Infrastructure (CNKI) by using “Chinese” and “teacher beliefs” as keywords. These papers were converted into txt format. Word segmentation, word frequency, and semantic analysis were then performed by means of the ROST CM6 software to obtain the semantic network among these key words (Figure 1).

From the semantic network connection strength in Figure 1, the connection strength of high-frequency words of CAL teacher beliefs research is clearly presented. Each node indicates a high-frequency word, and the interlaced lines between them represent the connection strength between each other. From an overall point of view, the nodes are not balanced, with some being dense and others sparse. The semantic network centers on “teacher,” followed by “teaching,” “Chinese,” “belief,” “learning,” “knowledge,” “student,” “language,” “classroom,” and “development,” etc. These words function as the “bridge” to realize the connection of the entire semantic network. The relationship between these words reveals the major topics of the research on CAL teacher beliefs, which can provide a reference for the development of the dimensions of the CAL teacher belief scale.

Table 1 reveals that the connection strengths of “Chinese” and “teacher,” “teacher” and “teaching” are among the highest, and the strength between “teacher” and “belief” is also as high as 1,867. Excluding the search keywords, the high-frequency words “Chinese” and “teaching” have the highest connection strength (2,152). The connection strength between “teaching” and “belief,” “teaching” and “research,” “classroom” and “teaching,” and “language” and “teaching” are 1,499, 1,347, 1,050, and 1,022, respectively. From the high semantic connection strength of “teaching” and other high-frequency words, it can be seen that “teaching” is one of the important dimensions of CAL teachers’ beliefs. In order to obtain good practical ability, teachers should conduct teaching research to ensure effective teaching in the classroom (Ismail et al., 2019). Therefore, the teaching-based research is of colossal necessity for effective CAL teaching, and the teaching belief is of top priority in the CAL teacher beliefs system.

**TABLE 1:** Semantic network connection strength.

From	To	Strength	From	To	Strength
Chinese	Teacher	2,816	Student	Teaching	1,078
Teacher	Teaching	2,737	Classroom	Teaching	1,050
Chinese	Teaching	2,152	Language	Teaching	1,022
Teacher	Belief	1,867	Ability	Teacher	1,018
Teacher	Research	1,682	Teacher	Knowledge	998
Teaching	Belief	1,499	Language	Teacher	979
Teaching	Research	1,347	Study	Teacher	950
Chinese	Belief	1,279	Study	Teaching	927
Student	Teacher	1,249	Chinese	Classroom	890
Classroom	Teacher	1,234	Chinese	Student	880
Chinese	Research	1,151	Teacher	Development	848

Besides, the connection strength between “Chinese” and “belief,” “Chinese” and “research,” “Chinese” and “class,” and “Chinese” and “student” decreasingly ranges from 1,279 to 844. It is indicated that CAL teachers need to be competent at Chinese ontology knowledge. The characteristics of Chinese language and the uniqueness of CAL teaching will affect teachers’ beliefs (Zhao, 2018). CAL teachers and researchers need to make arduous efforts to facilitate learners’ acquisition of Chinese language (Gong et al., 2020b). Therefore, *BCL* should not be ignored when exploring CAL teacher beliefs.

Furthermore, the connection strength of “student” with “teacher,” “student” with “teaching” and “Chinese” with “student” are 1,249, 1,078, and 880, respectively. Present classroom teaching attaches much importance to the teacher-student relationship. On the one hand, given that learning Chinese is challenging for many CAL learners, researchers should rethink the differences between CAL learners and native Chinese speakers (Gong et al., 2020b). On the other hand, CAL teachers are supposed to play diversified roles such as being knowledge imparter, organizer, helper, and instructor in the process of students’ knowledge construction in CAL class (Zhao, 2018). As teacher-student relationship bears a critical part of language teaching (Ang, 2005), it is therefore essential to include the beliefs about learners into the CAL teachers’ belief system.

Additionally, the connection strength of “ability” with “teacher,” “teacher” with “knowledge,” “study” with “teacher,” and “teacher” with “development” are 1,018, 998, 950, and 848, respectively. These high-frequency words are all related to CAL teacher’s beliefs about self-learning. Language teachers themselves hold certain beliefs about learning that will affect their teaching practice (Horwitz, 1985; Peacock, 2001). Hence, CAL teachers’ beliefs about self-learning are a crucial part of their belief system.

Based on the above analysis and by referring to previous studies (i.e., Song, 2018; Zhao, 2018; Zhu, 2019), the initial Pre-service CAL Teacher Belief Scale developed for this study comprised four dimensions: *Belief about Chinese Language Teaching*, *Belief about Chinese Language*, *Belief about Chinese Language Learners*, and *Belief about Self-learning*. These dimensions assonate Borg’s (2001) classification of teacher beliefs: belief of teaching, belief of learning, belief of learners, beliefs of subject knowledge, and belief of teacher roles. After determining the dimensions of the scale, this study further draws on the CAL belief scales by Song (2018), Zhao (2018), and Zhu (2019) to design specific items of this scale and preliminarily formulate the questionnaire.

Before the questionnaire survey was officially administered, a pilot study was conducted to examine the potential problems (Rong, 2010; Gan et al., 2019). The authors consulted a senior researcher who holds a doctorate in applied linguistics for suggestions on the development and revision of the scale. Then, eight students were invited to participate in the pilot study, including four undergraduates and four postgraduate students majoring in TCSOL, who were divided into two groups for discussion. Both teachers and students put forward some reasonable suggestions for the modification of this questionnaire. The specific modification suggestions are presented in Table 2. Following the feedback obtained from the pilot study, modifications were accordingly made until the scale was finalized.

**TABLE 2:** Modifications of the items after the pilot study.

Item (before)	Reasons for modification	Item (after)
Item 7: In CAL teaching, I will take a tolerant attitude toward Students’ mistakes.	The word “error” is vague, and the word “tolerance” is inappropriate.	Item 7: In CAL teaching, I will take an inclusive attitude toward Students’ inaccurate use of language.
Item 9: If students have different cognition levels, their learning effects in Chinese will also be different.	“Cognitive level” is too general and should be specific.	Item 9: If students have different language cognition levels, their learning effects in Chinese will also be different.
Item 12: Chinese is more complicated than other languages in the world.	“Languages in the world” is too broad.	Item 12: Chinese is more complicated than other languages commonly used in the world.
Item 16: I think the younger the student, the easier it is to learn a language.	The word “easier” is vague.	Item 16: I think the younger the students are, the more advantage they have in learning languages.

The Pre-service CAL Teacher Beliefs Scale (PCALTBS) includes two parts. Part One inquires the demographic information of the participants regarding their gender, grade of education, and age. Part Two contains 21 items of the Pre-service CAL Teacher Beliefs. It comprises four dimensions, namely, Pre-service CAL Teachers’ BCL (6 items: Item 1, 4, 6, 8, 12, 14), BCLT (5 items: Item 3, 5, 7, 11, 13), BCLL (5 items: Item 2, 9, 10, 15, 16), and Beliefs about Self-learning (5 items: Item 17, 18, 19, 20, 21). The questionnaire follows a 5-point Likert scale, ranging from “*Strongly Disagree* (1)” to “*Strongly Agree* (5).”

When responding to the questionnaire, the participants were first briefed about the objective of the study and answering methods. The respondents were guaranteed that the results of the survey would only be used for academic purposes, and their information would be kept confidentially. The survey was conducted in Chinese. It took 6–8 min for the participants to complete the questionnaire. 500 copies of the questionnaire were distributed. With the incomplete or wrongly answered copies removed, 443 valid copies of the questionnaire were obtained for further analysis.

The 443 valid copies constituted 253 undergraduates and 190 postgraduates majoring in TCSOL (Table 3). They were sequentially numbered into two groups with the odd numbers as Sample 1 (*N* = 221) and the even numbers as Sample 2 (*N* = 222). Sample 1 was used for EFA and Sample 2 for Confirmatory Factor Analysis (CFA). Sample 1 was composed of 22 males and 199 females, with an average age of 22.16. Sample 2 encompassed 25 males and 197 females, averagely aged 21.29. The number of students from each grade ranged from 31 to 48. The chi-square tests revealed that the two samples non-significantly distinguish from each other in terms of gender (*P* = 1.00 > 0.05), grade (*P* = 1.00 > 0.05), and age (*P* = 0.317 > 0.05).

**TABLE 3:** Demographic information of the participants.

Sample	Gender		Grade						Average age	Sum
	Male	Female	Freshman	Sophomore	Junior	Senior	PG year 1	PG year 2		
1	22	199	31	31	32	32	48	47	22.16	221
2	25	197	32	32	32	31	47	48	21.29	222
*P*	*P* = 1.00		*P* = 1.00						*P* = 0.317	443

### Phase 1: Exploratory factor analysis

SPSS26.0 was used to conduct promax rotations of principal components analysis on Sample 1. This method allows internal correlations among latent factors, so that principal components can be extracted and the construct validity of the scale can be tested (Devellis, 2003). Before performing the EFA, the applicability test of the scale was carried out. The KMO test value was 0.864 (>0.700). With regard to the Bartlett’s Test, the Approx. Chi-Square was 1599.532; the df was 210; and the significance probability of the Bartlett’s Test was 0.000 (<0.001), indicating that the sample was suitable for factor analysis. The first principal component analysis extracted 6 factors with eigen values greater than 1. Then, items (Item 14, 7, 3, 3, 15, and 11) with loadings below 0.4 were removed. The final round of principal component analysis extracted 3 factors. The three factors account for 35.752% (Factor 1), 11.081% (Factor 2), and 7.463% (Factor 3) of the total variance, and cumulatively explain 54.296% of the total variance (Table 4). The Cronbach’s Alpha of the scale was 0.839, with the ones for the three components ranging from 0.614 to 0.854. These results show that the scale had reasonably high internal consistency.

**TABLE 4:** Extraction sums of squared loadings.

Factor	Eigen value	% of variance	Cumulative %	Cronbach’s alpha
Factor 1	5.363	35.752	35.752	0.854
Factor 2	1.662	11.081	46.833	0.618
Factor 3	1.119	7.463	54.296	0.614

The correlations among these factors were shown in Table 5. The correlations between Factor 1 and Factor 2, Factor 1 and Factor 3, Factor 2 and Factor 3 are 0.504, 0.385, and 0.465, respectively. These correlation coefficients show moderately strong correlation among the factors and indicate that the scale had good discriminative validity (Gan et al., 2019). Furthermore, the correlations between the factors and the whole scale were all above 0.700, suggesting a high contribution rate of each factor and a satisfactory convergent validity. The result also confirms the appropriateness of the method of selecting oblique rotation (Sun et al., 2016).

**TABLE 5:** The correlation among the factors.

/	/	Factor 1	Factor 2	Factor 3	Scale
Factor 1	Pearson’s correlation coefficient	1			
	Sig.				
Factor 2	Pearson’s correlation coefficient	0.504**	1		
	Sig.	0.000			
Factor 3	Pearson’s correlation coefficient	0.385**	0.465**	1	
	Sig.	0.000	0.000		
Scale	Pearson’s correlation coefficient	0.844**	0.799**	0.736**	1
	Sig.	0.000	0.000	0.000	

**Correlation is significant at the 0.01 level (2-tailed).

The three factors were named as follows (Table 6): Factor 1 comprises 7 items (Item 5, 13, 17, 18, 19, 20, 21) with Cronbach’s Alpha of 0.854 (Table 4), which is reasonably high. The loadings of each item ranged from 0.568 to 0.876. These items are associated with pre-service CAL teachers’ *BCLT* experience, strategies, practice, and cognition. Therefore, this factor is labeled as *Pre-service CAL Teachers’ Beliefs about Chinese Language Teaching (BCLT)*.

**TABLE 6:** Result of exploratory factor analysis.

Factor	Item	Loading
Factor 1: Beliefs about Chinese language teaching	20 CAL teachers should combine teaching practice and research to improve their teaching and research capabilities.	0.876
	19 CAL teachers should always review and reflect on their own teaching experience and make improvements.	0.861
	21 CAL teachers should learn different knowledge across subjects in order to broaden their knowledge.	0.840
	18 CAL teachers should understand the new methods in the field of language education and apply the new methods to teaching practice.	0.818
	13 In international Chinese language teaching, I will take students as the center of the classroom.	0.588
	17 CAL teachers should work hard to learn different languages to improve their teaching ability.	0.576
	5 In international Chinese language teaching, I think teacher-student interaction is very important.	0.568
Factor 2: Beliefs about Chinese language	1 Chinese can be divided into characters, words, phrases and other elements.	0.751
	8 Chinese is a subject of knowledge.	0.645
	6 Chinese is combined according to certain grammatical rules.	0.544
	4 Chinese is a communication tool.	0.465
Factor 3: Beliefs about Chinese language learners	16 The younger the students, the more advantage they have in language learning.	0.734
	10 The language communication skills of extroverted students are stronger.	0.648
	12 Chinese is more complicated than other languages commonly used in the world.	0.554
	9 If students have different language cognition levels, their learning effects in Chinese will also be different.	0.497

Factor 2 subsumes 4 items (Item 1, 4, 6, 8). The Cronbach’s Alpha of this factor is 0.618, which is acceptable for quantitative studies in applied linguistics (Dörnyei, 2007; Ahn and Yang, 2009). The loadings of each item included in this factor spanned from 0.456 to 0.751. Since these items are related to the CAL teachers’ perception of Chinese language knowledge, this factor is thus titled as *Pre-service CAL Teachers’ Beliefs about Chinese Language (BCL)*.

Factor 3 encompasses 4 items (Item 9, 10, 12, 16) with Cronbach’s Alpha of 0.614. The loadings of each item range from 0.497 to 0.734. These items pertain to the CAL teachers’ understanding of the Chinese language learners. Therefore, this factor is termed as *Pre-service CAL Teachers’ Beliefs about Chinese Language Learners (BCLL)*.

### Phase 2: Confirmatory factor analysis

**FIGURE 2:**
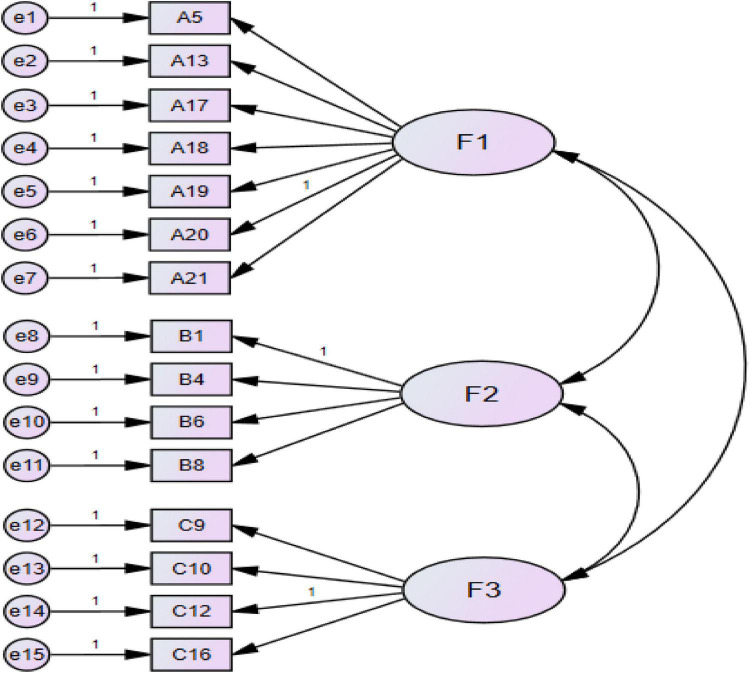
Initial model.

CFA was performed on Sample 2 by utilizing AMOS 24.0 to cross-validate the model established from the EFA exploration. The initial model based on the EFA factor model (Table 6) with Sample Two is shown in Figure 2. This path diagram clearly shows the factor structure of the theoretical conception, and each variable is only loaded on one factor.

**FIGURE 3:**
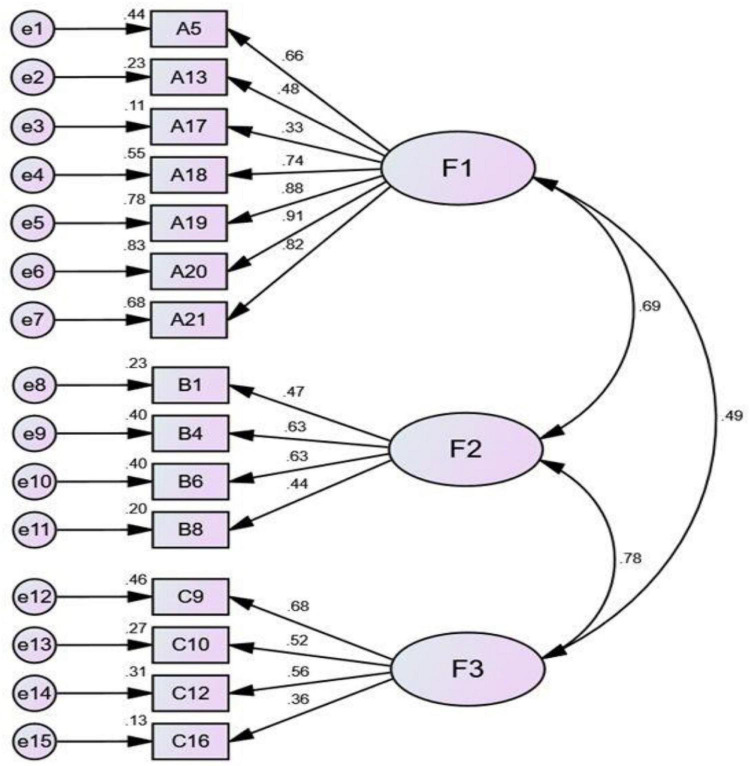
Model 1.

The path diagram of the first model is shown in Figure 3, and the fitting index is reported in Table 7. Generally speaking, the smaller the chi-square value, the better the model. However, there is no absolute standard, because the chi-square value will be affected not only by the number of samples, but also by the complexity of the model (Bentler and Bonett, 1980; Marsh and Hocevar, 1985; Marsh et al., 1988). The commonly suggested standard of χ^2^/df is ≤ 3.0 (Kline, 2015); GFI (Goodness of Fit Index) is ≥ 0.90, and AGFI (Adjust Goodness of Fit Index) is ≥ 0.90 (Bentler, 1983; Hu and Bentler, 1999); RMSEA (Root Mean Square Error of Approximation) is ≤ 0.08 (McDonald and Ho, 2002); NFI (Normed Fit Index) is ≥ 0.90 (Bentler and Bonett, 1980); IFI (Incremental Fit Index) is ≥ 0.90 (Zhang, 2011), and CFI (Comparative Fit Index) is ≥ 0.90 (Zhang, 2011). It can be seen that the fitting index of Model 1 in terms of χ^2^ (173.756), χ^2^/df (1.997), GFI (0.910), AGFI (0.876), RMSEA (0.067), IFI (0.927), and CFI (0.926) all meet the suggested criteria, but the index of NFI (0.864) is not satisfactory. The model thus needs to be modified. The modification indices of Model 1 show that the modification index of residual e3 and other residuals is as high as 12.180.

**TABLE 7:** Ideal model index and fitting parameters of the four models.

Model	χ ^2^	χ ^2^/df	GFI	AGFI	RMSEA	NFI	IFI	CFI	Deleted item
Suggested standard		≤3.0	≥0.9	≥0.8	≤0.08	≥0.9	≥0.9	≥0.9	
Model 1	173.756	1.997	0.910	0.876	0.067	0.864	0.927	0.926	17
Model 2	144.060	1.947	0.920	0.887	0.066	0.883	0.939	0.938	13
Model 3	120.884	1.950	0.927	0.892	0.066	0.895	0.946	0.945	16
Model 4	104.205	2.043	0.931	0.895	0.069	0.907	0.950	0.949	

**FIGURE 4:**
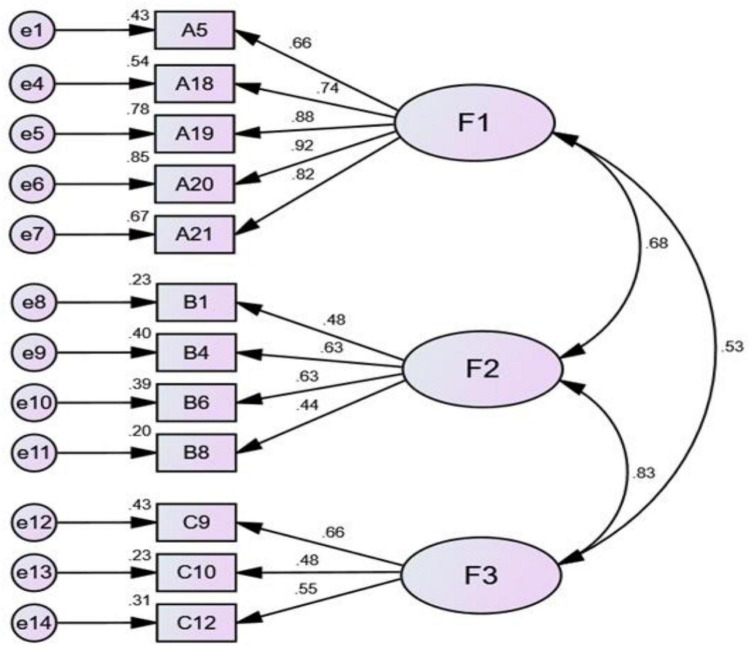
Model 4.

Modification indices can be used to transform a poorly fitting model into a well-fitting one by computing a score test with one degree of freedom for each fixed parameter in the model. For those parameters with large test statistics, the model can be iteratively re-estimated after lifting the relevant constraints until the desired level of fit is achieved. However, the practice of data-driven model modification has been widely criticized (Mansolf et al., 2020). Therefore, we decide to delete Item 17. After deleting Item 17, Model 2 is obtained. Compared with Model 1, the fitting parameters of Model 2 have been improved, but the NFI (0.864) has not yet met the suggested standard. The correction index of Model 2 reveals that the modification index of residual e2 is 7.570, which is slightly high. Therefore, Item 13 corresponding to residual e2 is deleted, from which Model 3 is obtained. Model 3 is still not up to the suggested standard. The path coefficient of residual e15 in the revised index of model 3 is as low as 0.13. Therefore, Item 16 corresponding to e15 is deleted, and Model 4 is then obtained. All parameters of Model 4 (Figure 4) now reach the suggested standard: χ^2^ is 104.205; χ^2^/df is 2.043 (<3.0); GFI is 0.931 (>0.9); AGFI is 0.895 (>0.8); RMSEA is 0.069 (<0.08); NFI is 0.907 (>0.9); IFI is 0.950 (>0.9) and CFI is 0.949 (>0.9).

After three rounds of revisions of the model indicators, and based on Raykov’s rho coefficient (Kline, 2015), the Composite Reliability of Model 4 is calculated. The Raykov’s rho coefficient for the three factors are 0.9036 (Factor 1), 0.6307 (Factor 2), and 0.5844 (Factor 3), respectively. These results suggest that the combined reliability of the three factors is reasonably satisfactory (Gan et al., 2019). The final Pre-service Chinese as an Additional Language Teacher Beliefs scale (PCALTBS) consists of 12 items (Table A1), covering *BCLT* (five items: Item 5, 18, 19, 20, 21), *BCL* (four items: Item 1, 4, 6, 8), and *BCLL* (three items: Item 9, 10, 12).

## Discussion

This study aims to develop and validate a scale for measuring the pre-service CAL teacher beliefs. After cross-validation by means of EFA and CFA, the final scale consists of 3 dimensions with 12 items, including *BCLT*, *BCL*, and *BCLL*, respectively.

The first factor (five items) identified in this study is labeled as “*BCLT.*” This subscale was aimed to assess the pre-service CAL teachers’ perception of Chinese language teaching in L2 class. This factor includes the pre-service CAL teachers’ beliefs about the importance of teacher-student interaction (Item 5), teaching pedagogies (Item 18), self-reflection on their teaching (Item 19), combining teaching practices with research (Item 20), and acquiring interdisciplinary knowledge (Item 21). The inclusion of these items under *BCLT* resonate the findings on grammar teaching beliefs of ESL/EFL teachers reported by Andrews (2003) and the teaching beliefs summarized by Lavigne (2014). Specifically, this is reflected in the items regarding the pre-service CAL teachers’ efforts to learn different languages, to adopt new teaching methods, to reflect on their teaching practice, to combine their teaching practice and research, and to acquire more knowledge for CAL teaching. It is postulated that the pre-service CAL teachers with high scores on this factor seem to attach copious importance to the methods and effects of Chinese language teaching practice, and have certain interest in CAL teaching research. They tend to be willing to try new methods, accept new knowledge, and constantly break through themselves.

The second validated factor (four items) is tagged as “*BCL.*” This subscale refers to the pre-service CAL teachers’ understanding of Chinese language. This is manifested in the items with regard to the pre-service CAL teachers’ beliefs about the elements of Chinese characters (Item 1), the communication nature of Chinese language (Item 4), grammatical rules (Item 6), and the nature of Chinese language as a subject of knowledge (Item 8). These items pertain to the pre-service CAL teachers’ perception of Chinese ontology knowledge, grammar and function in particular. This result corroborates Gong et al. (2020b) that CAL teachers are committed to exploring the domains of Chinese language in their teaching practice. It reveals that the pre-service CAL teachers evidently beware the significance of having sound knowledge of Chinese language. It is speculated that pre-service CAL teacher with higher scores on this factor have a certain grasp of the knowledge of Chinese ontology, and can correctly understand the characteristics and functions of Chinese language.

The third subscale (three items) for this study is named as “*BCLL.*” This factor was targeted at measuring the pre-service CAL teachers’ cognition of Chinese language learners in L2 class. It subsumes the items regarding the pre-service CAL teachers’ beliefs about the potential connection between learners’ cognition and learning efficiency (Item 9), the language communication skills of extroverted students (item 10), and the complexity of Chinese language to foreign learners (Item 12). These items associate with pre-service CAL teachers’ perspectives and attitudes toward Chinese language learners. The items, including the pre-service CAL teachers’ belief about “students of different language cognition levels” and “extroverted students with better communication skills,” suffices to illustrate this point. This finding echoes Gong et al.’s (2020a) conclusion that the background of the CAL learners should be paid with due attention in CAL class. It is posited that pre-service CAL teachers who score high on this factor have a certain degree of understanding of the characteristics of Chinese learners.

The three-dimensional PCALTBS developed in this study contributes to elucidate the construct and nature of pre-service CAL teacher beliefs. The three subscales consisting of *BCLT*, *BCL*, and *Beliefs about Chinese Learners* indicate both convergences and variations from those of other teacher populations. The items included in this study were originally adopted and modified from the items of previously developed scales aimed for picturing CAL teacher beliefs (Zhu, 2019) and CAL volunteer teacher beliefs (Song, 2018). This result thus suggests the consistency of the pre-service CAL teacher beliefs with those of other CAL teacher populations. More importantly, the three elements constructed in this study could reflect the features specific to the pre-service CAL teachers. The PCALTBS in this study was developed by extracting the high-frequency words of pre-service CAL teachers’ beliefs reported in previous studies. It could thus reflect the features unique to pre-service CAL teachers.

The PCALTBS scale developed in this study accommodates the shortage of instruments aimed to measure CAL teacher beliefs, pre-service CAL teacher beliefs in particular. To date, there are a limited number of studies on the beliefs of pre-service CAL teachers. The few existing scales for measuring pre-service CAL teacher beliefs were primarily adopted from the scales intended for ESL/EFL teachers’ beliefs. For instance, the pre-service CAL teachers’ grammar teaching beliefs scale (Ji and Liu, 2012) is adapted from Andrews (2003) ESL teachers’ grammar teaching beliefs scale. Although there are some similarities between teaching English as an Additional Language (EAL) and teaching Chinese as an Additional Language (CAL), the scales for CAL teachers’ beliefs adapted from the ones for ESL/EFL teachers are likely to lack the applicability to and uniqueness of the CAL teachers’ beliefs. In light of the discrepancy between teaching EAL and teaching CAL, the PCALTBS developed in this study thus provides a context-specific instrument for gauging the CAL pre-service teachers’ beliefs.

The scale validated in this study sheds light on the techniques utilized to develop quantitative instruments for measuring CAL teacher beliefs. The development of teacher beliefs scales reported in previous studies tends to mainly rely on the means of literature review either by listing previous research results or by constructing the dimensions of the scale based on subjective perception (Song, 2018; Zhu, 2019). These methods might lend certain theoretical support to the justification of the scales later developed. However, instruments designed out of such techniques may lack empirical evidence. Besides a thorough review of the relevant literature, the present study resorted to semantic analysis by means of ROST-CM6 to screen out high-frequency words and their connection strengths in related studies when deciding the dimensions of pre-service CAL teachers’ beliefs scale. Hence, the dimensions of the PCALTBS in this study could reflect the hotspots of previous studies on CAL teachers’ beliefs, conform to the characteristics of CAL teachers’ beliefs, and thus ensure solid validity of the instrument.

Additionally, the PCALTBS scale developed in this study goes beyond the existing scales for quantifying CAL teacher beliefs by presenting solid and scientific verification of reliability and validity. Little previous research reported use of such techniques as EFA and CFA to verify the reliability and validity of its instrument to quantitatively measure beliefs of CAL teachers (Zhu, 2019). Often is found with a report of the Cronbach’s Alpha and KMO value, which probably fails to guarantee the reliability and validity of the scales (e.g., Song, 2018). Auxiliary research in the form of semi-structured interviews and classroom observations is thus suggested to be applied as alternative ways (Altan, 2012; Jaimes, 2013; Çapan, 2014). Though triangulation might be achieved by such means, few scales in the field of applied linguistics could generate reliable and valid results (Dörnyei, 2007). Therefore, better verifications such as EFA and CFA could to a large degree ensure the reliability and validity of a scale.

Nevertheless, it should be acknowledged that the Cronbach’s Alphas of BCL (0.618) and BCLL (0.614) as reported in this study were not that perfectly high. This may be expounded by the fact that the number of items is one of the factors that shall contribute to the Cronbach’s Alpha (Dörnyei, 2007; Ahn and Yang, 2009). The BCL includes 4 items and BCLL only contains 3 items. Therefore, this might explain the reasons why these two factors only displayed acceptable but not perfect Cronbach’s Alphas. In a similar vein, the RMSEA value reported in CFA was 0.67, which only achieved the acceptable level but not excellent (Xia and Yang, 2019). This is probably due to the fact that degree of freedom was only 51, and the sample size of CFA model was relatively small as well. When degree of freedom and sample size are large, the RMSEA will decrease (Kenny et al., 2015).

In summary, this study has validated that the PCALTBS can be used as a reliable scale to measure pre-service CAL teachers’ belief. The PCALTBS scale for the present study showcases the standard procedure for developing and validating a scale. Although belief is a complex construct, scrupulous analysis of specific beliefs with appropriate methods and considerate research designs can make research on beliefs feasible and effective (Pajares, 1992). First of all, the PCALTBS has undergone a rigorous pilot study, and then EFA was carried out through Sample 1, and three factors were extracted to test the reliability and structural validity of the scale. Then, grounded on the EFA results, CFA was carried out on Sample 2. It is assumed that the three factors together constituted a potential factor: pre-service CAL teachers’ belief, based on which a model was constructed. After three rounds of modifications, the results of Model 4 showed that the model fitting parameters of the revised scale reached the suggested criteria of ideal indicators, and the construct validity was ideal. The three-factor structure of the final scale was supported by the data, indicating that pre-service CAL teachers’ belief is a three-dimensional system consisting of *BCLT*, *BCL*, and *Beliefs about Chinese Learners*. Therefore, PCALTBS is proved to be a reasonable construct for the pre-service CAL teachers’ belief system, and can effectively measure the pre-service CAL teachers’ belief. The instrument developed and validated in the present study enriches but does not replace the already existing scales for measuring CAL teacher beliefs, particularly pre-service CAL teacher beliefs.

## Conclusion

By means of semantic analysis of extracting high-frequency words and the semantic connection values between those words relating to beliefs of CAL teachers, this study developed and validated the Pre-service Chinese as an Additional Language Teacher Belief Scale (PCALTBS). Following the standard steps of psychometrics on the preparation of the scale, and after the cross-validation of EFA and CFA, the PCALTBS with 12 items was finally obtained. The PCALTBS has been found with good psychometric properties. It is verified that the pre-service CAL teachers’ belief is a multi-dimensional construct, including *BCLT*, *BCL*, and *BCLL*. This study provides empirical evidence for developing and validating an instrument of measuring pre-service CAL teacher beliefs. It compensates for the lack of self-developed and reliability-validated instruments in research on beliefs of CAL teachers, pre-service CAL teachers in particular. The development and validation of the PCALTBS standardizes the measuring process of pre-service CAL teacher beliefs and could reasonably elucidate their beliefs.

The PCALTBS of this study offers pedagogical implications for sustainable development of pre-service CAL student teachers, instructors and institutions. From the pre-service CAL teachers’ perspective, an education in the field of TCSOL means potential orientation in their future employment upon graduation (Wang and Zhang, 2021). The scale validated in this study could function as an instrument for pre-service CAL teachers to understand their beliefs system, which could facilitate them to make rational judgment on whether they are suitable to be a CAL teacher or not. Besides, in addition to professional courses in the pre-service study stage, attention is also invited from the pre-service CAL teachers to establishing appropriate beliefs and cultivating enthusiasm for CAL teaching. A positive beliefs system could nurture deep commitment to CAL teaching. With regard to instructors, the PCALTBS can help them sensitize the beliefs system of the pre-service CAL teachers, through which pre-service CAL teacher trainers could get deeper understanding of the pre-service CAL teachers’ professional curriculum learning situation and their future employment orientations. It is advisable for pre-service CAL instructors and teacher trainers not to only physically impose the professional knowledge onto the student teachers, but to attach greater importance to their mental needs as a potential TCSOL practitioner. A training program that could effectively help the pre-service CAL teachers nurture positive attitudes and beliefs toward teaching shall be conducive for them to establish their identities in this field. As for institutions, the PCALTBS indicates directions for sustaining pre-service CAL teacher development. Through the measurement of pre-service CAL teachers’ beliefs, institutions can promptly diagnose problems in the training process and seek countermeasures. In addition, institutions are suggested to actively cooperate with international schools, foreign universities, primary and secondary schools, and online CAL teaching organizations so as to build multi-dimensional CAL teaching practice and employment platforms for pre-service CAL teachers, which could ensure the employment rate of TCSOL students and accordingly enhance their confidence in the prospect of CAL teaching career.

Although the results demonstrate that the PCALTBS can be a reliable instrument to gauge pre-service CAL teachers’ beliefs, there are some limitations. Firstly, the Cronbach’s Alpha coefficient for 2 factors (BCL and BCLL) is not that high. This may be explicated by the fact that the number of items is one of the factors that shall determine the Cronbach’s Alpha (Dörnyei, 2007; Ahn and Yang, 2009). Another limitation is related to the size of the sampled population. The sample of the study is relatively small, with only 443 respondents surveyed. Future research could be conducted to further validate the PCALTBS with a larger number of participants. More research is also suggested to utilize the PCALTBS to analyze the relationship between teacher beliefs and other variables, for obtaining a broader insight into the profiles of pre-service CAL teacher beliefs for the goal of sustaining development of CAL teachers.

## Data availability statement

The raw data supporting the conclusions of this article will be made available by the authors, without undue reservation.

## Ethics statement

The studies involving human participants were reviewed and approved by the School of Foreign Languages, Hubei University of Technology, China. The patients/participants provided their written informed consent to participate in this study.

## Author contributions

CL designed the study and finalized the draft. TY collected, analyzed, and made the first draft of the manuscript. SZ helped process the data. CM analyzed and helped revise the manuscript. HL helped revise the manuscript. All authors contributed to the article and approved the submitted version.

## Appendix

**APPENDIX TABLE A1:** The finalized items of the pre-service CAL teacher beliefs scale (PCALTBS).

Factor 1 Beliefs about Chinese language teaching
A5 In international Chinese language teaching, I think teacher-student interaction is very important.
A18 CAL teachers should understand the new methods in the field of language education and apply the new methods to teaching practice.
A19 CAL teachers should always review and reflect on their own teaching experience and make improvements.
A20 CAL teachers should combine teaching practice and research to improve their teaching and research capabilities.
A21 CAL teachers should learn different knowledge across subjects in order to broaden their knowledge.
**Factor 2 Beliefs about Chinese language**
B1 Chinese can be divided into characters, words, phrases, and other elements.
B4 Chinese is a communication tool.
B6 Chinese is combined according to certain grammatical rules.
B8 Chinese is a subject of knowledge.
**Factor 3 Beliefs about Chinese language learners**
C9 If students have different language cognition levels, their learning effects in Chinese will also be different.
C10 The language communication skills of extroverted students are stronger.
C12 Chinese is more complicated than other languages commonly used in the world.
